# Cluster-based analysis improves predictive validity of spike-triggered receptive field estimates

**DOI:** 10.1371/journal.pone.0183914

**Published:** 2017-09-06

**Authors:** James Bigelow, Brian J. Malone

**Affiliations:** 1 Coleman Memorial Laboratory, University of California, San Francisco, California, United States of America; 2 Department of Otolaryngology—Head and Neck Surgery, University of California, San Francisco, California, United States of America; 3 Kavli Institute for Fundamental Neuroscience, University of California, San Francisco, California, United States of America; University of Oldenburg, GERMANY

## Abstract

Spectrotemporal receptive field (STRF) characterization is a central goal of auditory physiology. STRFs are often approximated by the spike-triggered average (STA), which reflects the average stimulus preceding a spike. In many cases, the raw STA is subjected to a threshold defined by gain values expected by chance. However, such correction methods have not been universally adopted, and the consequences of specific gain-thresholding approaches have not been investigated systematically. Here, we evaluate two classes of statistical correction techniques, using the resulting STRF estimates to predict responses to a novel validation stimulus. The first, more traditional technique eliminated STRF pixels (time-frequency bins) with gain values expected by chance. This correction method yielded significant increases in prediction accuracy, including when the threshold setting was optimized for each unit. The second technique was a two-step thresholding procedure wherein clusters of contiguous pixels surviving an initial gain threshold were then subjected to a cluster mass threshold based on summed pixel values. This approach significantly improved upon even the best gain-thresholding techniques. Additional analyses suggested that allowing threshold settings to vary independently for excitatory and inhibitory subfields of the STRF resulted in only marginal additional gains, at best. In summary, augmenting reverse correlation techniques with principled statistical correction choices increased prediction accuracy by over 80% for multi-unit STRFs and by over 40% for single-unit STRFs, furthering the interpretational relevance of the recovered spectrotemporal filters for auditory systems analysis.

## Introduction

Receptive field characterization is fundamental to sensory physiology. In recent decades, the spectrotemporal receptive field (STRF) has emerged as a preferred model for representing stimulus features that drive neurons throughout the auditory pathway [1–7]. Analogously, spatial-temporal receptive fields have been widely used to characterize responses of visual [8–11] and somatosensory neurons [12–13]. As its name implies, the STRF summarizes the joint spectral (spatial) and temporal features that evoke dynamic changes in the firing rates of sensory neurons. STRFs may also be used to identify stimulus features that evoke subthreshold neuronal responses [14–15], or drive changes in the collective activity of cell populations, such as those reflected in multi-unit action potential recordings [4,16–18], local field potentials [19–20], and electrocorticography signals [21–23]. STRFs are frequently incorporated with an output nonlinearity (e.g., to account for response threshold and saturation) into the Linear-Nonlinear encoding model that can be used to generate predicted responses to novel input [24].

The most common method for estimating STRFs in the auditory system is the spike-triggered average (STA), which comprises the average stimulus preceding a spike [1,6,24–28]. High gain regions of the STA are thought to reveal the stimulus subspace filtered by the neuron, i.e., the subset of stimulus dimensions that evoke a neuronal response. Although the STA can theoretically yield a rigorous characterization of a neuron’s linear stimulus-response transformation, practical limitations are assumed to introduce some degree of noise into STRF estimates obtained with this methodology [6]. For instance, constraints on recording time naturally impose finite boundaries on the stimulus space that can be sampled, and limit the number of spikes that can be obtained for calculation of the STA. Thus, even with a fully balanced stimulus ensemble, stimulus features falling outside a neuron’s receptive field typically fail to cancel out perfectly in the STA, resulting in nonzero gain values, assumed to reflect measurement error, scattered throughout the putatively nonresponsive regions of probed STRF space. For this reason, most studies of STRF structure have implemented one of various corrective procedures in an effort to more closely approximate the true underlying STRF [6–7,29–35]. One classic correction technique, still widely used in the auditory STRF literature [17,22,36–38], involves identifying statistically significant regions of the STRF simply by zeroing time-frequency bins (pixels, for digital STRF representations) with gain values expected by chance [17,22,26,31,36–40]. Chance values can be determined from null STAs computed with randomized spike times [31], and gain threshold choices usually correspond to values of the null distribution with conservative (*p* < 0.01–0.001) probability values [17,22,26,31,36–40].

Statistical correction choices may be especially important for applications requiring highly dimensional STRFs. For instance, neurons throughout the primate auditory pathway are capable of both exquisitely fine spectral selectivity (~0.1 octave scale [41–43]) and temporal response precision (~millisecond scale [44–48]), particularly in alert subjects [42]. At the same time, many neurons exhibit broad spectral and/or temporal integration properties, especially in hierarchically-advanced stations such as auditory cortex, where spectral bandwidths spanning multiple octaves and temporal integration times exceeding 100 ms are common [6,49]. Capturing the response properties of such heterogeneous neuronal populations thus requires both broad bandwidth and high spectrotemporal resolution. Under the gain thresholding technique described above, the likelihood of false positive (Type I) errors increases with the number of pixels in the STRF [50–52]. For example, even a null STA with 200 × 200 time/frequency bins is expected to have 400 “significant” pixels after gain thresholding at the *p* < 0.01 significance level. Adopting a more conservative threshold would limit the false positives, but increase false negative (Type II) errors. Such a compromise between sensitivity and specificity is well known in the functional magnetic resonance imaging (fMRI) literature, where analyses routinely include many thousands of voxels [50–52]. The most popular approach to this large-scale multiple testing problem is a two-step, cluster-based correction procedure [51,53–54]. Clusters of contiguous voxels surviving an initial activation threshold are then subjected to a cluster-extent (or mass) threshold, such that only clusters exceeding a specified number of voxels (or summed activation value) are retained for subsequent analysis.

Although numerous investigations have concluded that cluster-based correction appropriately balances false positive and false negative errors in fMRI studies, similar approaches are rare the receptive field estimation literature [26,55]. Moreover, most STRF studies implementing gain thresholding techniques as described above tend to adopt conventional significance thresholds (*p* < 0.01–0.001), without further examining the consequences of these choices. To our knowledge, neither gain-thresholding nor cluster-analysis techniques have been formally evaluated in the STRF literature, e.g., in terms of performance-based metrics such as accuracy in predicting responses to novel stimuli [32–34]. Thus, the analyses in the present study were structured around two primary objectives: first, to systematically investigate the consequences of specific gain thresholding settings on resulting STRF structure, and second, to test the utility of two-step, cluster-based correction procedures for improving STRF validity. We approached these objectives by applying a range of liberal to conservative gain and cluster-based thresholds to raw STAs obtained from awake primate auditory cortex. The validity of each threshold setting was then evaluated by comparing observed neuronal responses elicited by a novel validation stimulus to the predicted response obtained using each version of the corrected STRF [29,32–34,56–57]. We then extended these correction approaches by implementing a simple cross-validation heuristic for selecting the best threshold settings for individual STRFs. Finally, we investigated whether the foregoing approaches could be improved upon by independently selecting thresholds for the excitatory and inhibitory subregions of the STRF. In general, we find that prediction accuracy improves significantly following correction with conventional gain thresholding techniques, and that further meaningful improvements were only possible with cluster-based correction techniques. Excitatory and inhibitory subfield versions of these approaches offered, at best, only marginal additional improvement.

## Materials and methods

### Subjects and surgical preparation

All procedures were carried out in strict compliance with recommendations in the Guide for the Care and Use of Laboratory Animals of the National Institutes of Health, and were approved by the Institutional Animal Care and Use Committee of the University of California, San Francisco. Details regarding protocol and methodology have been published previously [58–59]. A brief description follows.

Physiological data were collected from two adult squirrel monkeys (*Saimiri sciureus*, Monkey 1: male; Monkey 2: female). Subjects were group housed with other conspecifics in a temperature and humidity controlled colony. Subjects had *ad libitum* access to water and primate diet supplemented with fresh fruits and vegetables. An environmental enrichment program was administered by UCSF Laboratory Animal Resource Center staff. Regular monitoring and care was provided by UCSF veterinary staff.

Prior to physiological recording, subjects were trained to sit in a primate chair. A head post was then surgically implanted to allow head restraint. For all surgical procedures, subjects were sedated with ketamine (25 mg/kg) and midazolam (0.1 mg/kg), and anesthetized with isoflurane gas (0.5–5%). Implants were secured to the cranium with bone screws and dental acrylic. Perioperative antibiotics and analgesics were administered as needed in consultation with UCSF veterinary staff. After subjects were trained to sit in the primate chair while head fixed, they underwent a second surgery in which a recording chamber was implanted over primary auditory cortex (A1). The temporal muscle was resected, the cranium overlying auditory cortex was exposed, and a recording chamber was secured with bone screws and dental acrylic. Perioperative care was administered as before.

Sterile procedures were used for all recording sessions to access auditory cortex. Following lidocaine (1%) application, a small cranial burr hole (2–3 mm) was drilled inside the recording chamber under magnification with a surgical microscope. A small incision was then made in the dura using micro-surgical instruments. The process was repeated as needed for subsequent recording sessions to expose additional areas of auditory cortex. Between recording sessions, implants were cleaned aseptically and the chamber was filled with antibiotic ointment and sealed with a metal cap.

### Electrophysiology

Recordings were conducted inside a sound attenuation chamber (Industrial Acoustics Company, Bronx, NY). Extracellular data were collected using 16-channel linear electrode arrays (177 μm^2^ contact size, 150 μm spacing; NeuroNexus Technologies, Ann Arbor, MI). Probes were advanced into cortex with a hydraulic microdrive (David Kopf Instruments, Tujunga, CA) to depths at which neural activity was evident on most or all channels. Penetrations were approximately perpendicular to the surface of the exposed cortex, although it was not possible to achieve strict orthogonality for every recording given the complex anatomy of auditory cortex near the superior temporal sulcus [60–61]. Extracellular signals were amplified with an RA16 Medusa preamplifier (Tucker-Davis Technologies, Gainesville, FL), band-pass filtered (800–5000 Hz) and stored to hard disk at 30.3 kHz using a Cheetah A/D system (Neuralynx, Inc., Bozeman, MT) for offline analysis. Spike waveforms that exceeded three median absolute deviations of the raw voltage distribution were retained for further analysis.

Both multi-unit (MU) and isolated single-unit (SU) signals were analyzed. Custom MATLAB software (MathWorks, Natick, MA) was used for spike waveform detection, outlier rejection, and sorting. Template matching was used in combination with manual sorting in 2D and 3D waveform feature space (e.g., projections onto principal components, peak/valley amplitude, spike times). Autocorrelation, cross-correlation, and refractory period analyses were used to support SU classifications. Only SUs that remained active for the duration of the recording were included in subsequent analyses. Filtered spikes that could not be assigned to a SU were considered MU signals.

### Stimulus presentation

Sounds were delivered through a free-field speaker directly in front of the subject, 40 cm from the interaural line. Sound levels were calibrated using a Brüel & Kjær Model 2209 meter using an A-weighted decibel filter and a Model 4192 microphone. Levels were constrained across the experiment between 64 and 66 dB, and sound levels within the same recording session fell within 1 dB of each other.

**Fig 1:**
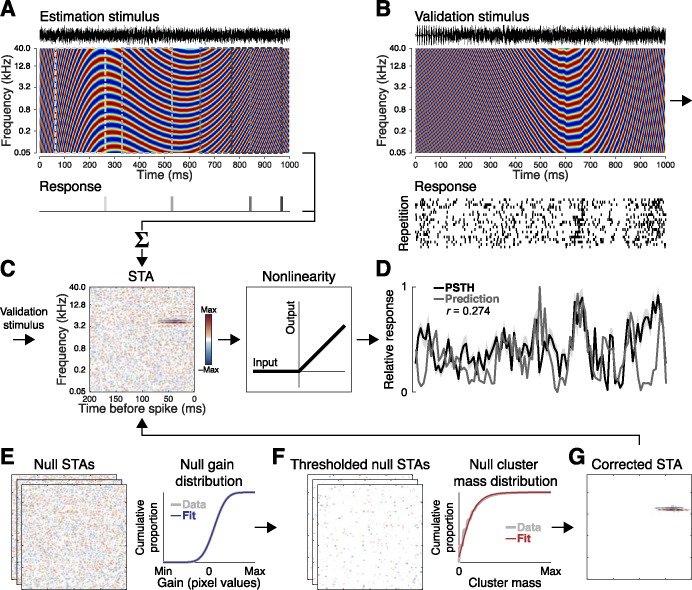
Spectrotemporal receptive field (STRF) estimation and validation procedures. **(A)** Units were first probed with a 30-min dynamic moving ripple (DMR), a synthetic broadband stimulus sharing many features with natural sounds including local spectrotemporal correlations. **(B)** Responses elicited by a novel 30-s DMR segment (50 repetitions) were used for subsequent validation and testing. **(C)** STRFs were estimated by calculating the spike-triggered average (STA). Response predictions were obtained by convolution of the STRF and validation spectrogram, with the output nonlinearity modeled by half-wave rectification. **(D)** STRF validity was assessed by calculating the correlation coefficient between predictions and neuronal responses obtained with the trial-averaged peristimulus time histogram (PSTH). **(E)** Null STAs computed with circularly-shifted spike times were used to generate a sample of gain values expected by chance. A normal distribution fit to these values was used to determine gain value cutoffs corresponding to a logarithmically-spaced range of significance levels from *p* < 10^0^ to *p* < 10^−9^. **(F)** Similarly, null STAs subjected to a gain threshold were used to generate a sample of cluster mass values expected by chance, and a gamma distribution fit to these values was used to identify cluster mass cutoffs corresponding to the same range of significance levels. **(G)** The corrected STA was defined by pixels (clusters) exceeding a specified significance level.

The stimulus used for estimating STRFs (below) was a dynamic moving ripple (DMR; Fig 1A), which has been extensively used in auditory STRF analysis as described in detail elsewhere [5–6,31,39]. Briefly, the DMR is a temporally-varying broadband stimulus that shares many features with natural sounds such as short-term (local) spectrotemporal correlations, but is fully balanced in the long term for durations exceeding a few minutes [31]. It is thus capable of driving auditory cortical responses, and permits rigorous STRF estimates using the STA method without additional correction for stimulus correlations. For the present experiment, the duration of the DMR was 30 min and comprised ~40 sinusoidal carriers per octave spanning 50–40,000 Hz, each with randomized phase. Carrier magnitude was modulated by the spectrotemporal envelope, which at a given time is defined by a single spectral (peaks/oct) and temporal modulation rate (peaks/s). The spectral modulation rate varied from 0–4 cycles per octave, and the temporal modulation rate varied between –150 Hz (upward sweep) and 150 Hz (downward sweep). Both modulation parameters varied randomly and independently over time, and were statistically independent and unbiased within their respective ranges. Maximum modulation depth was 40 dB with a logarithmic amplitude distribution [39]. A unique 30-s DMR segment (50 repetitions), generated with the same parameters as the estimation stimulus, was used as a validation stimulus to assess the prediction accuracy of the STRF estimates (Fig 1B).

### STRF analysis and validation

All analyses were performed in MATLAB (MathWorks, Natick, MA). Raw STAs were obtained for each unit by computing the average stimulus preceding each spike (Fig 1C). STRFs were estimated at a resolution of 193 frequency bins (~0.05 oct, y-axis) and 200 time bins (1 ms, x-axis) to adequately reflect the spectral and temporal encoding fidelity of the auditory system in awake primates [41–48]. The color spectrum (z-axis) used to define STRF pixel values corresponds to the spike rate relative to the mean, such that red and blue reflect firing rates above or below the mean, respectively [31]. For simplicity, we refer to these responses as the excitatory and inhibitory regions of the STRF, respectively [17]. We use the term *gain* to refer to the strength of these responses (reflected in STRF pixel intensity values).

Neuronal responses evoked by the validation stimulus were evaluated by computing trial-averaged peristimulus time histograms (PSTHs). These were compared to predicted responses (Fig 1D) obtained by convolution of the STRF with the validation stimulus (MATLAB conv2 function; [16,23,40,62]). Half-wave rectification was used as a simple approximation of the output nonlinearity characteristic of extracellular recordings [40,62]. The correlation coefficient between the response and prediction was used to quantify prediction accuracy. As described in further detail below, this stimulus was randomly divided into two 15-s halves, one for validation and the other for testing.

### Statistical correction approaches

Raw STAs were corrected with a broad continuum of liberal-to-conservative statistical thresholds corresponding to 30 logarithmically spaced significance values ranging from *p* = 10^0^ to *p* = 10^−9^ (note that *p* = 10^0^ reflects the uncorrected STA). For each STA, gain threshold cutoff values corresponding to each *p* value (expressed *p*_gain_) were obtained by fitting a normal distribution to a sample of null STA gain values (Fig 1E). Expressed in this way, each gain threshold corresponds to the proportion of the null distribution with values exceeding the specified *p* value. For example, *p*_gain_ < 0.01 denotes that only STRF pixels with gain values exceeding the most extreme 1% of the null distribution were retained for further analysis. To obtain the null STAs, spike times were circularly shifted by a random value (MATLAB circshift function) selected from an interval equal to the stimulus duration [63]. Thus, if spikes near the beginning of the stimulus were shifted toward the middle of the stimulus, spikes near the end of the stimulus wrapped around to the beginning. An STA was then computed with the shifted spike times using the same axes and resolution as the true STA (200 iterations). The circular shifting approach ensured that both spike counts and inter-spike interval (ISI) distributions were preserved across the true and null STAs. The validity of the STRFs obtained with each gain threshold setting was then evaluated in terms of prediction accuracy.

A similar approach was implemented in a two-step correction procedure comprising a gain threshold followed by a cluster-based threshold. Following gain thresholding, the remaining clusters (contiguous pixels identified with the MATLAB bwconncomp function) were subjected to a range of cluster mass thresholds corresponding to the same 30 logarithmically spaced *p* values described above. Cluster mass was defined as the summed absolute pixel values. Cluster mass cutoffs corresponding to each *p* value were obtained for each STA by fitting a gamma distribution to a sample of null clusters (Fig 1F). For computational efficiency, the null cluster mass distribution was obtained from the sample of 200 null STAs by computing the masses of clusters remaining in every *i*th null STA after applying gain thresholds computed with every *j*th null STA. The same range of gain thresholds described above was applied to STAs for subsequent cluster analysis with the exception of the most extremely liberal and conservative settings, as follows: [i] The *p* value reflecting the raw STA (*p* = 10^0^) was omitted since no gain threshold was implemented, and thus, no clusters were available for analysis, [ii] The *p* value reflecting the most liberal gain threshold (*p* < 0.49) was omitted because it generally produced only a small number of extremely large clusters, [iii] The eight most conservative gain threshold settings (approximate range: *p* < 10^−7^ to 10^−9^) were omitted because such extreme gain thresholds applied to null STAs typically resulted in very few or zero surviving clusters. Thus, STAs were first corrected at the pixel (gain) level with a total of 20 thresholds (approximate range: *p* < 0.24 to 10^−6^), and subsequently corrected at the cluster (mass) level with 30 thresholds reflecting the original range of *p* values (10^0^ to 10^−9^). STRF structure resulting from each gain and cluster threshold intersection (expressed *p*_(gain,clst)_) was evaluated in terms of prediction accuracy as described above.

Although previous studies have applied the same significance thresholds to excitatory and inhibitory regions of the STRF [31], it is conceivable that STRF validity could benefit from independent thresholding. This is because, as numerous studies have reported, inhibitory regions tend to be less robust and stereotyped, and more variable than excitatory regions of the STRF [11,17,30,64]. Thus, an excitation-dominated STRF might benefit from more rigorous elimination of inhibitory pixels and clusters. To test this hypothesis, the correction approaches outlined above were extended to two additional analyses in which excitatory and inhibitory pixels and clusters were thresholded independently. This yielded a total of four statistical correction approaches which are summarized and compared below: [1] pixel gain correction (*p*_gain_), [2] independent excitatory and inhibitory pixel gain correction (*p*_gain{exc,inh}_), [3] cluster mass correction (*p*_(gain,clst)_), [4] independent excitatory and inhibitory cluster mass correction (*p*_(gain,clst{exc,inh})_). We note that, contrary to our *a priori* expectations, independent excitatory and inhibitory subfield analysis yielded little, if any, significant improvement in predictive validity over the more basic approaches. As such, all figures pertaining to these analyses are presented in the Supporting Information section (S1–S4 Figs) to permit better focus on the principal results of the paper.

Two implementations of each of the foregoing gain- and cluster-based thresholding approaches are summarized in the results below. First, a fixed-parameter approach was tested in which all units were uniformly subjected to the same threshold settings. Each unit was exhaustively tested at all possible intersections of the significance levels included in our study (*p* < 10^0^ to *p* < 10^−9^). For each unit, this yielded a vector of 30 prediction correlation values for gain thresholding alone (*p*_gain_), a matrix of 30 × 30 prediction values for independent excitatory and inhibitory gain thresholding (*p*_gain{exc,inh}_), a matrix of 20 × 30 prediction values for gain- plus cluster-thresholding (*p*_(gain,clst)_), and an array of 20 × 30 × 30 values for independent excitatory and inhibitory cluster mass correction (*p*_(gain,clst{exc,inh})_). For the second approach, best threshold settings were selected for each unit via cross-validation. To avoid overfitting these threshold settings, the validation stimulus was randomly divided into two equal 15-s segments. The threshold settings that maximized prediction accuracy for the first half of the data (validation dataset) were then evaluated in terms of prediction accuracy for the second half (test dataset). For the fixed-parameter approaches and raw STA, prediction values reflect the test dataset alone. To minimize the dependence of the results on any particular definition of the validation and test datasets, the procedure was repeated ten times for randomly-selected dataset halves. Prediction correlation values reported below indicate the mean across iterations.

Because the present study was primarily concerned with the comparative consequences of gain and cluster correction choices, no smoothing was applied to responses, predictions, or STRF kernels. Unless otherwise noted, all analyses included the full estimation, validation, and test datasets. To ensure the results reflected units with reliable responses during both the estimation and validation phases, each multi-unit and single-unit was characterized with the reliability index (RI [22]) and trial similarity (TS [65]) metrics. To calculate RI, the estimation dataset was first divided into 30 1-min segments. An STRF was then computed using half of the segments selected at random (*p*_gain_ < 0.05), and a second STRF was computed using the remaining segments. RI was defined as the mean correlation coefficient between the two STRFs across 200 iterations. TS was computed by constructing a PSTH from half of the validation trials selected at random (bin size = 10 ms), and a second PSTH from the remaining trials. TS was defined as the mean correlation coefficient between PSTHs across 100 iterations. The RI and TS calculations were repeated using circularly-shifted spike times, and only units with RI and TS values exceeding chance levels (*p* < 0.01) were retained for subsequent analysis. Unit populations were not screened further, e.g., by prediction significance criteria. To facilitate comparison with previous studies, the results focus on responses and predictions analyzed at 10-ms resolution. Additional summaries are provided for bin sizes of 1, 2, 5, 10, 20, 50, and 100 ms.

### Temporal and spectral modulation preferences

Modulation properties of each unit were obtained by computing the two-dimensional Fourier transform of each version of the corrected STRF, as described in detail elsewhere [17,31,66]. Briefly, the Fourier transform is a function of temporal (-150 to 150 cycles/s) and spectral modulation frequency (0 to 4 cycles/octave). The ripple transfer function (RTF) is obtained by folding along the temporal midline (temporal modulation frequency = 0). Summing down the columns of the RTF yields the temporal modulation transfer function (tMTF), and summing across the rows of the RTF yields the spectral modulation transfer function (sMTF). MTFs were considered band-pass if values above and below the peak of the MTF decreased by at least 3 dB. All others were considered low-pass (high-pass MTFs were not encountered). The best modulation frequency (BMF) was defined as the peak of the MTF for band-pass MTFs, and the mean between zero and the 3-dB upper cutoff for low-pass MTFs.

## Results

Recordings from 66 probes were included in the study: 50 from Monkey 1 (21 left hemisphere) and 16 from Monkey 2 (6 left hemisphere). A total of *n* = 354 multi-units and *n* = 289 single-units satisfied RI and TS criteria described above and were retained for further analysis. Adopting more liberal unit inclusion criteria produced results similar to those reported below, but yielded lower raw mean prediction accuracy values and greater prediction improvements following STRF correction (data not shown). Thus, the results reported below conservatively represent prediction improvements following STRF correction. Qualitatively similar outcomes were observed for MU and SU STRFs. However, because significant quantitative differences were obtained in many of the analyses below, the results for each data type are reported separately.

Consistent with previous studies [11,17,30,64], STRFs in our sample exhibited a significant excitatory bias (S1 Fig). The mean ratio between excitatory and inhibitory pixels with the largest gain values was 1.83 for MU and 1.60 for SU STRFs (MU: *p* < 10^−41^, SU: *p* < 10^−31^; Wilcoxon signed-rank tests). Similarly, the mean ratio of summed excitatory and inhibitory cluster mass values, after applying a gain threshold at *p*_gain_ < 0.05, was 1.21 for MU and 1.16 for SU STRFs (MU: *p* < 10^−34^, SU: *p* < 10^−20^; Wilcoxon signed-rank tests).

### Gain thresholding

**Fig 2:**
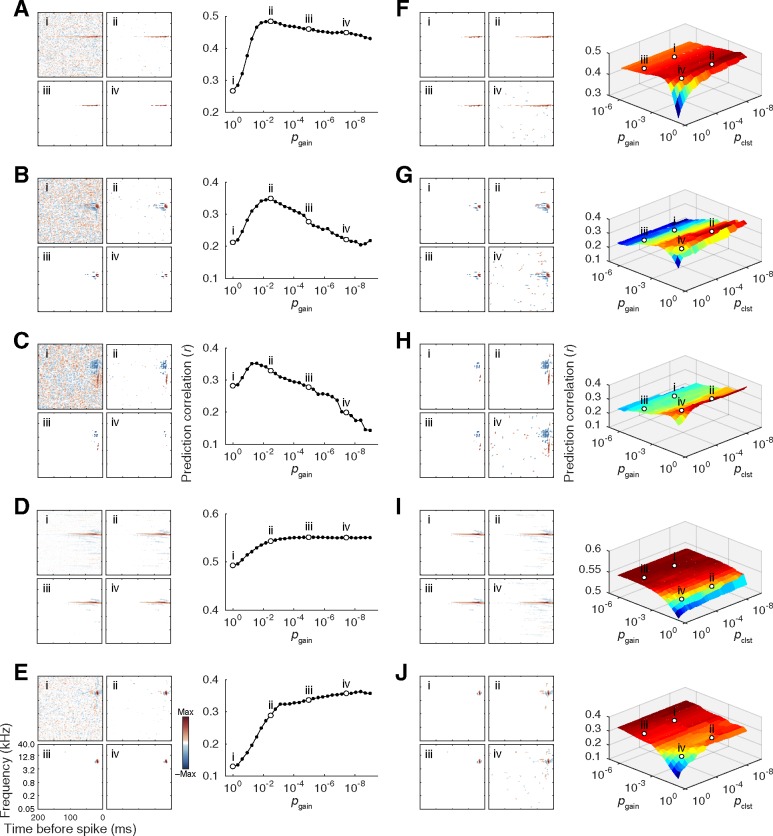
Example data depicting results of STRF gain and cluster-mass thresholding procedures. Raw STAs were corrected with a continuum of liberal-to-conservative gain and cluster-mass thresholds, expressed in terms of chance probability (*p*_gain_, *p*_(gain,clst)_) determined by null STA values computed with randomly shifted spike times. The same five example units are used to illustrate the consequences of gain thresholding (**A–E)** and cluster-based analysis (**F–J)**. For each subplot, corrected STAs are shown on the left, and plots of prediction accuracy at each tested threshold setting are shown on the right (note: *p*_gain_ = 10^0^ and *p*_(gain,clst)_ = 10^0^ reflect the raw STA). Typically, prediction accuracy increased with moderate gain thresholding but decreased for more stringent settings **(A–B)**, in some cases, falling below values obtained with the raw STA **(C)** For other units, prediction accuracy plateaued **(D)** or continued to increase **(E)** with increasingly stringent correction. For the cluster-based approach, contiguous pixels surviving an initial gain threshold were subjected to cluster-mass thresholding based on summed absolute values of clusters obtained from null STAs. For most units, peak prediction accuracy was obtained using a relatively liberal gain threshold followed by a more stringent cluster-mass threshold **(F–H)**. although in some cases, prediction accuracy benefited primarily from gain thresholding alone **(I)**, or increased asymptotically at symmetrically stringent settings for both thresholds **(J)**.

**Fig 3:**
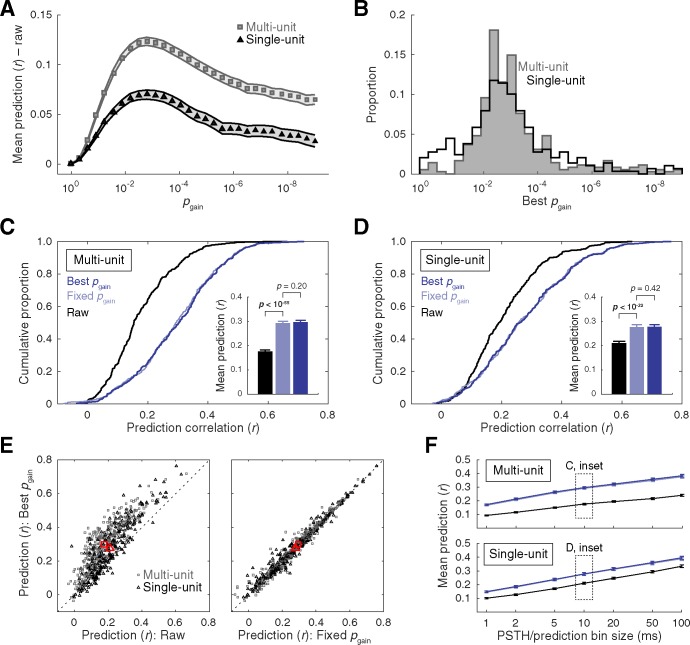
Summary of STRF gain thresholding results. **(A)** Mean ± SEM prediction accuracy at each gain threshold (*p*_gain_) minus prediction accuracy obtained with the raw STA (MU: *r* = 0.176, SU: *r* = 0.210). **(B)** Histograms of gain thresholds that yielded the highest prediction accuracy. **(C–D)** Cumulative distribution functions of prediction accuracy obtained with raw STAs, STRFs corrected with a fixed *p*_gain_ setting (*p*_gain_ < 0.01 applied uniformly across units), and STRFs corrected at the best *p*_gain_ settings (identified on an individual-unit basis through cross-validation). Inset bar plots represent mean +SEM prediction accuracy. For both data types, accuracy for each gain thresholding approach (fixed *p*_gain_ and best *p*_gain_) was significantly higher than the raw STA, but these approaches significantly did not differ from each other (Wilcoxon signed-rank tests). **(E)** Scatter plots of prediction accuracy for individual units at best *p*_gain_ versus raw (left) and fixed *p*_gain_ (right). Note that prediction accuracy may be higher for the fixed *p*_gain_ setting than best *p*_gain_ setting. This is because best *p*_gain_ settings determined by the validation dataset may not maximize prediction accuracy for the test dataset (see Methods for additional details). Red markers indicate the means. **(F)** Mean ±SEM prediction accuracy as a function of the temporal bin size for the PSTHs and predictions using the same correction approaches and color schemes as in **(C–D)**.

The consequences of statistical thresholding choices were first investigated by correcting raw STAs with a continuum of liberal-to-conservative pixel gain thresholds. Prediction accuracy was then calculated for each of the corrected STRFs to assess their validity. Example data depicting the results of the correction procedure are provided in Fig 2, and a summary is presented in Fig 3. Mean prediction accuracy obtained with the raw STAs was *r* = 0.176 for the MU sample and *r* = 0.210 for the SU sample, a significant difference (*p* < 10^−3^; Wilcoxon rank-sum test). Gain thresholding improved prediction accuracy for the majority of STRFs, with mean prediction accuracy at the best *p*_gain_ setting increasing to *r* = 0.297 and *r* = 0.278 for MU and SU data, respectively. The improvement was highly significant for both data types (MU: *p* < 10^−57^, SU: *p* < 10^−32^; Wilcoxon signed-rank tests), and significantly higher for the MU sample (*p* < 10^−25^; Wilcoxon rank-sum test). Most units reached peak prediction accuracy within the range of approximately *p*_gain_ < 0.01 to *p*_gain_ < 0.001 (Fig 3A and 3B). In the interest of placing these results in the context of previous studies, prediction accuracy was directly compared between the units’ best *p*_gain_ settings (identified on an individual-unit basis through cross-validation) and a fixed setting of *p*_gain_ < 0.01 (applied uniformly across units), chosen to represent a conventional gain threshold [24,30–33]. As seen in Fig 3C and 3D, prediction accuracy was significantly higher at the fixed *p*_gain_ < 0.01 setting than raw (MU: *p* < 10^−55^, SU: *p* < 10^−32^; Wilcoxon signed-rank tests), and statistically equal to the units’ best *p*_gain_ setting (MU: *p* = 0.20, SU: *p* = 0.42; Wilcoxon signed-rank tests). This finding implies that, on average, it was possible to capture virtually all of the improvement afforded by the *p*_gain_ optimization heuristic by adopting a simpler fixed setting of *p*_gain_ < 0.01. Similar outcomes were obtained at alternative temporal discretizations of the predictions and responses, with a general trend toward increasing prediction accuracy at larger bins (Fig 3F).

### Independent excitatory and inhibitory gain thresholding

To assess whether the foregoing results could be improved upon by independent gain thresholding of the excitatory and inhibitory regions of the STRF, prediction accuracy was assessed for all possible intersections of 30 excitatory and 30 inhibitory gain thresholds corresponding to the *p* values used in the original gain thresholding procedure. Prediction accuracy was then evaluated at the units’ best *p*_gain{exc,inh}_ and *p*_gain_ settings (chosen by cross-validation), as well as at a conventional fixed setting of *p*_gain{exc,inh}_ < 0.01. Example data are shown in S2 Fig, and the results are summarized in S3 Fig. In general, the best *p*_gain{exc,inh}_ thresholds fell within a similar range to the best *p*_gain_ settings (S3A–S3D Fig). A significant bias toward more conservative best *p*_gain{inh}_ thresholds was detected in the MU sample (*p* < 10^−10^; two-sample K-S test), whereas a trend toward the same direction in the SU sample did not reach significance (*p* = 0.62; two-sample K-S test). As seen in S3E and S3F Fig, neither the fixed *p*_gain{exc,inh}_ < 0.01 setting nor the units’ best *p*_gain{exc,inh}_ settings substantially improved upon mean prediction accuracy obtained with the best *p*_gain_ threshold. For the MU data, prediction accuracies were *r* = 0.297 for the best *p*_gain_ setting, *r* = 0.293 for the fixed *p*_gain{exc,inh}_ < 0.01 setting, and *r* = 0.298 for the best *p*_gain{exc,inh}_ setting; none of the pairwise comparisons reached significance (*p* > 0.10, Wilcoxon signed-rank tests). For the SU data, prediction accuracies were *r* = 0.278 for the best *p*_gain_ setting, *r* = 0.277 for the fixed *p*_gain{exc,inh}_ < 0.01 setting, and *r* = 0.276 for the best *p*_gain{exc,inh}_ setting. The difference between the best *p*_gain_ and fixed *p*_gain{exc,inh}_ settings was not significant (*p* = 0.324, Wilcoxon signed-rank test), and the small decreased observed in the best *p*_gain{exc,inh}_ setting relative to the fixed *p*_gain{exc,inh}_ and best *p*_gain_ settings reached borderline significance (*p* = 0.030 and *p* = 0.003, respectively, Wilcoxon signed-rank tests). As above, similar outcomes were observed at smaller and larger bin sizes, with increasing prediction accuracy at larger bins (S3H Fig).

### Cluster-mass thresholding

**Fig 4:**
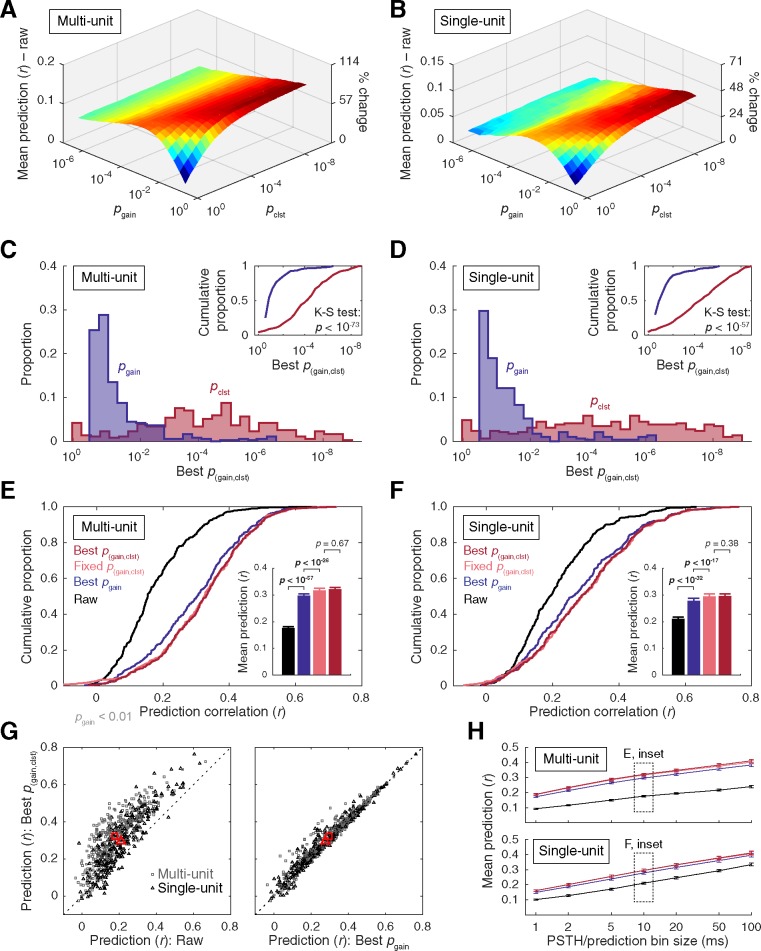
Summary of results from two-step (pixel-gain and cluster-mass) thresholding. **(A–B)** Mean prediction accuracy at each gain and cluster threshold intersection (*p*_(gain,clst)_) minus prediction accuracy obtained with raw STRFs (MU: *r* = 0.176, SU: *r* = 0.210). **(C–D)** Histograms of gain and cluster thresholds that yielded the highest prediction accuracy. Best *p*_clst_ settings were significantly more conservative than best *p*_gain_ settings for both data types (two-sample K-S tests). **(E–F)** Cumulative distribution functions of prediction accuracy obtained with raw STAs, STRFs corrected at the best *p*_gain_ settings (chosen for each unit by cross validation), STRFs corrected at a fixed setting (*p*_gain_ < 0.05, *p*_clst_ < 10^−5^ applied uniformly across units), and STRFs corrected at the best *p*_(gain,clst)_ settings (chosen for each unit by cross validation). Inset bar plots represent mean +SEM prediction accuracy. The fixed and best *p*_(gain,clst)_ settings yielded significant increases in prediction accuracy over the best *p*_gain_ method, and were statistically equivalent to each other (Wilcoxon signed-rank tests). **(G)** Scatter plots of prediction accuracy for individual units at best *p*_(gain,clst)_ versus raw (left) and best *p*_gain_ (right). Red markers indicate the means. **(H)** Mean ±SEM prediction accuracy as a function of the temporal bin size for the PSTHs and predictions using the same correction approaches and color schemes as in **(E–F)**.

The effectiveness of two-step (pixel- and cluster-based) thresholding was evaluated following the same general approach as the preceding gain correction procedures. Prediction accuracy was assessed at each intersection of gain and cluster mass thresholds detailed above. Example results are provided in Fig 2, and a summary in Fig 4. Prediction accuracy was generally highest at relatively liberal pixel gain threshold, followed by a more stringent cluster mass threshold (Fig 4A–4D). The distributions of best *p*_gain_ and *p*_clst_ threshold settings were significantly different for both data types (MU: *p* < 10^−73^, SU: *p* < 10^−57^; two-sample K-S tests). Prediction accuracy at the units’ best *p*_(gain,clst)_ settings improved to *r* = 0.321 and *r* = 0.295 for the MU and SU data, respectively (Fig 4E and 4F), a significant increase over predictions obtained with the best *p*_gain_ (MU: *p* < 10^−30^, SU: *p* < 10^−17^; Wilcoxon signed-rank tests) and best *p*_gain{exc,inh}_ settings (MU: *p* < 10^−27^, SU: *p* < 10^−20^; Wilcoxon signed-rank tests). As with the gain thresholding approaches, results obtained with the best *p*_(gain,clst)_ settings (chosen by cross-validation) were compared to a fixed-parameter approach in which the same gain and cluster thresholds were uniformly applied across units. Prediction accuracy obtained with conventional threshold settings fixed at *p*_(gain,clst)_ < 0.01 fell short of that obtained with the best *p*_(gain,clst)_ settings chosen for each unit. Mean accuracy for the fixed *p*_(gain,clst)_ < 0.01 setting was *r* = 0.309 for MU data and *r* = 0.286 for SU data, significantly lower than the best *p*_(gain,clst)_ values reported above (MU: *p* < 10^−7^, SU: *p* < 10^−4^; Wilcoxon signed-rank tests). Considering the asymmetric distributions of best *p*_gain_ and *p*_clst_ thresholds identified above, we next evaluated a fixed parameter approach in which the gain threshold setting was made more liberal (*p*_gain_ < 0.05) and the cluster threshold was made more conservative (*p*_clst_ < 10^−5^). As see in Fig 4E and 4F, mean accuracy obtained with this alternative version of the fixed *p*_(gain,clst)_ threshold approximated that obtained with the best *p*_(gain,clst)_ approach. Mean accuracy for the fixed *p*_gain_ < 0.05, *p*_clst_ < 10^−5^ setting was *r* = 0.317 for MU data and *r* = 0.295 for SU data, statistically equivalent to the best *p*_(gain,clst)_ values reported above (MU: *p* = 0.668, SU: *p* = 0.381; Wilcoxon signed-rank tests), and significantly greater than the best *p*_gain_ approach (MU: *p* < 10^−26^, SU: *p* < 10^−17^; Wilcoxon signed-rank tests). Similar outcomes were obtained for all bin sizes, with increasing prediction accuracy at larger bins (Fig 4H).

The comparative benefits of the cluster-based methodology over the more traditional gain-thresholding approach can be directly observed in Fig 4G (right panel), where change in performance is indicated by the vertical distance of the data points from the unity line. Consistent with mean values reported above, the majority of these data points fall above the unity line, implying superior cluster-based performance for these units. Nevertheless, some data points fell very near the unity line, suggesting equivalent performance for these units, and a minority fell below the unity line, implying that cluster-based correction was disadvantageous for these units. Visual inspection of this plot indicates most of the units with lower accuracy in the cluster-thresholding condition started out with very low prediction values (*r* < 0.1) in the gain-thresholding condition. Further, an approximately equal number of data points in this range fell above the unity line, raising the possibility that such STRFs may have been highly noisy to begin with, and thus could not be substantially improved by either correction method. For the remainder of STRFs, the largest benefits of cluster-methods were observed in the intermediate range of prediction values (*r* ≈ 0.1–0.4) following gain thresholding. Very little improvement was observed for units with high prediction correlations (*r* > 0.5). This tendency toward declining improvement (cluster-based prediction minus gain-based prediction) with increasing gain-based prediction accuracy was supported by a significant negative correlation for MU data points (r = -0.281, *p* < 10^−7^), and a trend in the same direction for the SU data (r = -0.094, *p* = 0.11). Considered together, these results raise the possibility that cluster-based methods may be most advantageous for moderately noisy STRFs with lower predictive power.

### Independent excitatory and inhibitory cluster-mass thresholding

For the final correction approach in our study, gain and cluster mass thresholds were implemented as above, with the exception that inhibitory and excitatory cluster mass thresholds were permitted to vary independently of one another. The effects of these independent thresholds can be seen in example data presented in S2 Fig, which are depicted at the gain threshold that yielded the highest mean prediction accuracy. Results are summarized in S4 Fig. Best *p*_clst{exc}_ and *p*_clst{inh}_ thresholds did not significantly differ from each other (MU: *p* = 0.61, SU: *p* = 0.15; two-sample K-S tests). A small but statistically reliable increase in prediction accuracy was observed between the best *p*_(gain,clst)_ and *p*_(gain,clst{exc,inh})_ settings (S4E and S4F Fig), from *r* = 0.321 to *r* = 0.326 for the MU data, and from *r* = 0.295 to *r* = 0.300 for the SU data (MU: *p* < 10^−7^, SU: *p* < 10^−5^; Wilcoxon signed-rank tests). Prediction accuracy obtained with the best *p*_(gain,clst{exc,inh})_ settings was also higher than a fixed parameter approach similar to the one adopted in the *p*_(gain,clst)_ method (*p*_gain_ < 0.05, *p*_clst{exc}_ < 10^−5^, *p*_clst{inh}_ < 10^−5^ applied uniformly across units). Mean accuracy for the fixed *p*_(gain,clst{exc,inh})_ setting was *r* = 0.316 for MU data and *r* = 0.292 for SU data, significantly lower than the best *p*_(gain,clst{exc,inh})_ approach (MU: *p* < 10^−3^, SU: *p* = 0.003; Wilcoxon signed-rank tests), and statistically equivalent to the best *p*_(gain,clst)_ approach (MU: *p* = 0.739, SU: *p* = 0.990; Wilcoxon signed-rank tests). Results were similar across bin size, with increasing prediction accuracy at larger bins (S4H Fig).

### Stimulus-driven variance explained

**Fig 5:**
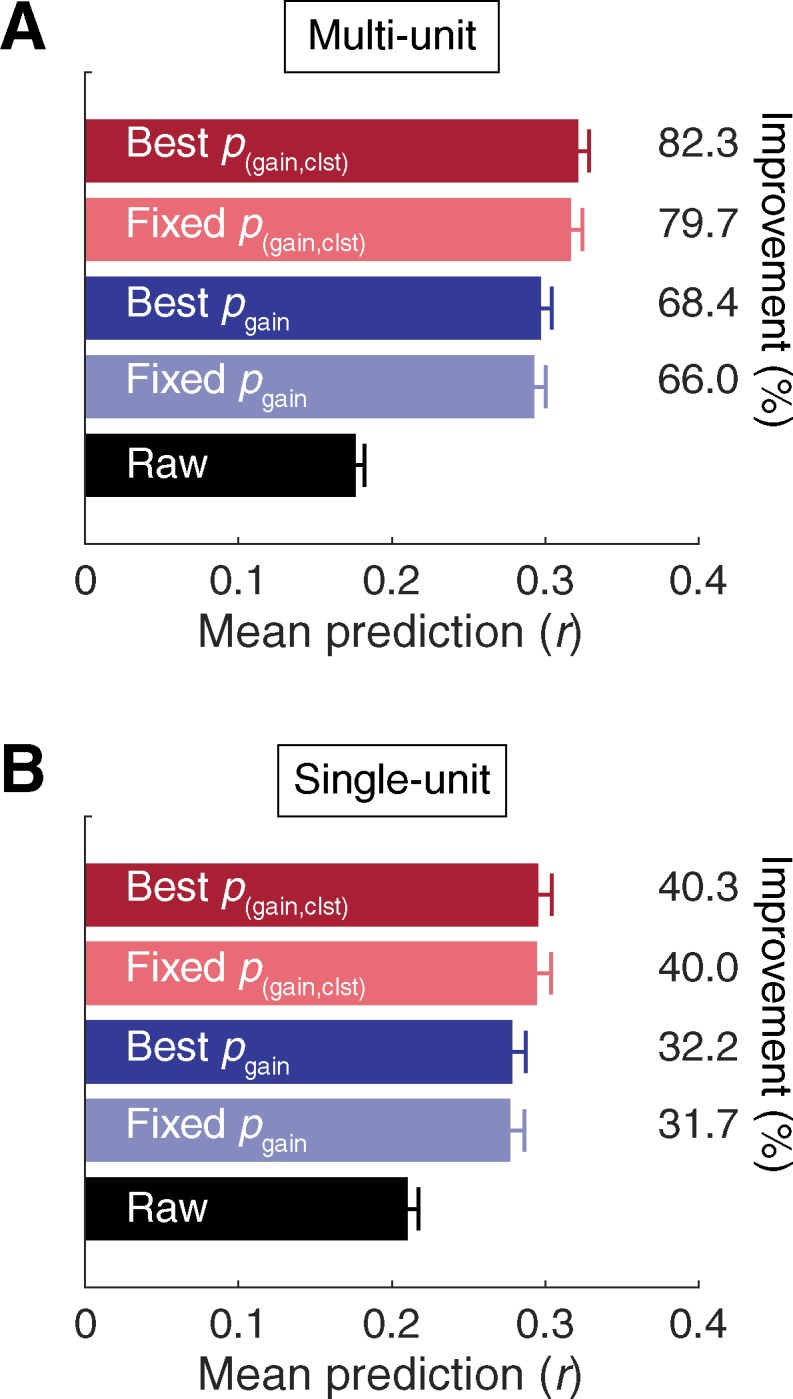
Summary of results from each statistical correction approach. Bar plots depict mean +SEM prediction accuracy for the **(A)** MU and **(B)** SU data samples. Percent improvement over raw (uncorrected STA) was calculated as *r*_corrected_−*r*_raw_ / *r*_raw_ * 100.

**Fig 6:**
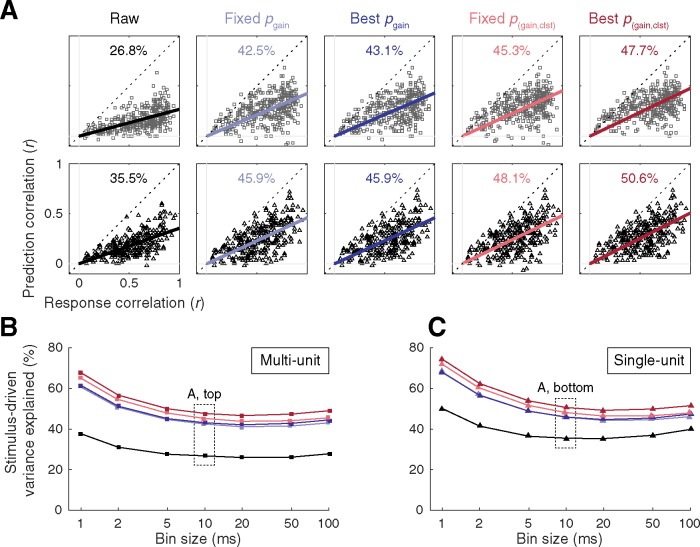
Stimulus-driven variance captured by STRFs corrected with each statistical thresholding approach. **(A)** Scatter plots summarizing the relationship between response correlations and prediction correlations obtained with STRFs using each statistical correction approach. Predictions were typically higher for units with reliable responses, however, even the highest prediction correlations generally fell short of the response correlations. The bold, colored lines represent the linear relation between response and prediction correlations, and the slopes of these lines can be used to estimate the proportion of stimulus-driven variance captured by each version of the STRFs. Top: Multi-unit. Bottom: Single-unit. **(B–C)** Estimates of stimulus-driven variance captured by STRF predictions were stable for bin size choices of 5 ms or larger, and increased using smaller bin sizes.

For convenience, mean prediction accuracy values obtained for each data type and each correction approach in our study are summarized in Fig 5. Mean prediction accuracy ranged from 0.176 (raw) to 0.321 (best *p*_(gain,clst)_) in the MU sample, and from 0.210 (raw) to 0.295 (best *p*_(gain,clst)_) in the SU sample. These values are comparable to results obtained in awake ferret auditory cortex [67], and are somewhat higher than a previous study in awake primate auditory cortex [68]. In general, STRF prediction accuracy in auditory cortex tends to be relatively poor compared to lower auditory structures such as inferior colliculus, where nonlinearities and contextual influences are less dominant, and responses to a repeated stimulus are more stereotyped [68–70]. As discussed elsewhere [56,71–73], intertrial response variability imposes an upper limit on prediction accuracy that can be obtained with a linear STRF model. This is because the activity of units with distinctive responses across trials is principally governed by factors other than the stimulus (e.g., contextual effects, top-down influences, measurement noise). Thus, the proportion of stimulus-driven variance captured by STRFs corrected with each statistical thresholding approach was estimated by computing the slope of the linear (least squares) relation between response and prediction correlations [73]. A slope near 0.5 would imply that predictions tended to be higher for units with reliable responses, but that only ~50% of the stimulus-driven variance in the PSTH was explained by the STRF prediction. For this analysis, two PSTHs were constructed using random halves of the validation trials. Response correlations were defined by the correlation between the two PSTHs (i.e., the TS metric [65]) and prediction correlations were defined by the correlation between one of the PSTHs and the prediction (mean across 100 iterations). As seen in Fig 6A, the proportion of stimulus-driven variance captured by the predictions was approximately one third using the raw STAs, and gradually increased to approximately one half using increasingly sophisticated correction procedures. Similar estimates were obtained with bin size choices of 5 ms or larger, and were generally higher at the smaller bin sizes (Fig 6B and 6C).

### Consequences of statistical correction on STRF structure

**Fig 7:**
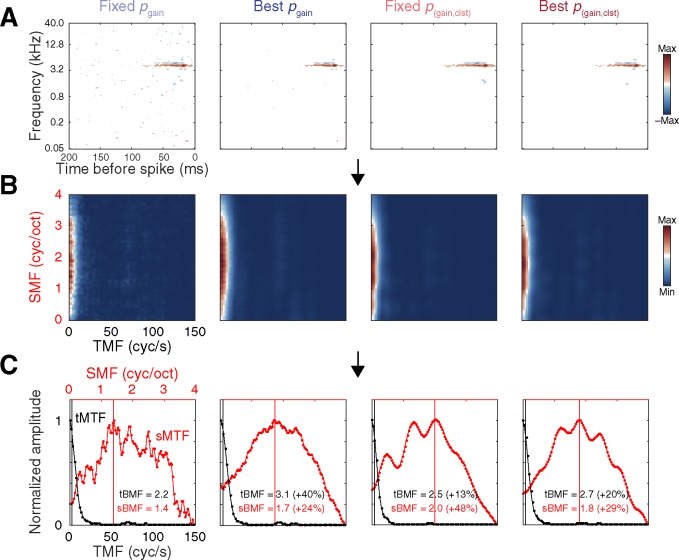
Example data depicting the influence of each statistical thresholding approach on STRF structure, as indexed by changes in temporal and spectral modulation preference estimates. **(A)** STRF estimates obtained with each correction approach. **(B)** RTFs obtained by computing the two-dimensional Fourier transform of each version of the STRF, and folding along the temporal midline. **(C)** tMTFs and sMTFs obtained by summing down the columns and across the rows of the RTF, respectively. Vertical lines indicate tBMFs and sBMFs. The percentages in parentheses indicate BMF differences relative to conventional thresholding (fixed *p*_gain_ < 0.01) obtained with each of the alternative correction approaches, calculated as [BMF_corrected_−BMF_conventional_] / BMF_conventional_ * 100.

**Fig 8:**
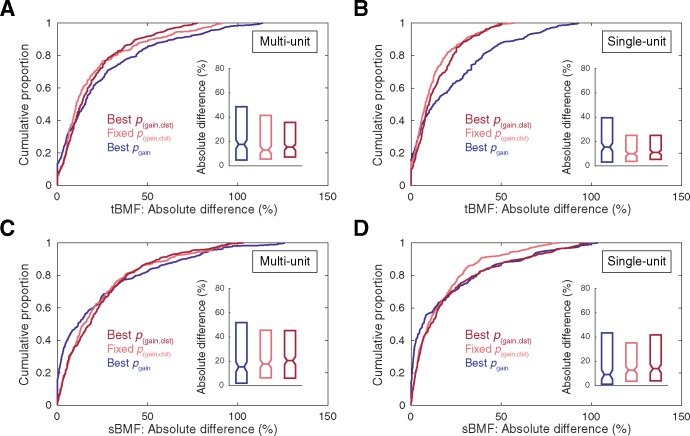
Summary of the consequences of statistical thresholding approaches for temporal and spectral modulation preference estimates. Absolute percent differences in parameter estimates (relative to values obtained with the conventionally-defined significant STRF) for tBMF **(A–B)** and sBMF **(C–D)**. For clarity, statistical outliers are omitted from the cumulative distribution plots (values exceeding the third quartile plus 1.5 times the inner quartile range).

The significant differences in prediction correlations reported above imply structural differences among STRFs obtained with each correction approach. It is important to note that any such differences arising during the initial receptive field estimation stage will carry over into all subsequent characterizations of receptive field structure, possibly giving rise to shifts in parameter estimates such as spectral/temporal preferences and bandwidths. To illustrate this point and provide additional context for understanding the consequences of statistical correction choices on STRF structure, spectral and temporal modulation preferences were estimated from RTFs obtained from each version of the corrected STRF. Because RTFs are typically obtained from statistically significant STRFs [17,31,66], the raw STA was omitted from this analysis and the conventionally-defined significant STRF (i.e., the fixed *p*_gain_ < 0.01 setting) was used as a baseline comparison for the remaining correction approaches. Structural differences were quantified by calculating the absolute percent change in BMF estimate relative to conventional ([BMF_corrected_−BMF_conventional_]/ BMF_conventional_ * 100). Examples of RTFs, MTFs, and BMF estimates obtained using each version of the corrected STRF are depicted in Fig 7, and population summaries are provided in Fig 8. For clarity, statistical outliers were omitted from the cumulative distribution plots in Fig 8 (values exceeding the third quartile plus 1.5 times the inner quartile range). As can be seen in the inset boxplots in Fig 8, BMF estimates obtained using non-conventional correction approaches typically changed by approximately 15% (range of median values across correction approaches and unit types: tBMF, 10.00–17.75%; sBMF, 9.09–20.66%).

## Discussion

Although receptive field characterization has been a central focus of sensory physiology for over a century [74], estimation methods–including error correction procedures–have not become standardized. Diverse approaches have appeared in the recent receptive field estimation literature [6–7,32–34], including methods that bypass error correction procedures altogether [68–69]. Simple gain-thresholding techniques applied to the STA continue to be widely used in the auditory STRF literature [17,22,36–38], likely in part because of their straightforward implementation and interpretation. In the present study, we validate the use of such gain thresholds for improving the predictive power of the raw STA. Significant improvements in mean prediction accuracy were obtained by adopting a uniform, conventional gain threshold (*p*_gain_ < 0.01) representative of numerous previous studies [17,22,36–38]. Indeed, we found that optimizing gain threshold settings for individual STRFs via cross validation failed to significantly improve upon this conventional approach (Fig 3). This was true even for a more refined optimization procedure in which excitatory and inhibitory gain thresholds were selected independently (S3 Fig). A central finding of our study, however, was that cluster-based analysis permitted meaningful improvement over these conventional and optimized gain-thresholding techniques (Figs 4 and 5). Most of the benefit was captured by selecting absolute gain and cluster-mass thresholds through cross validation, or by adopting fixed-parameter settings inspired by the asymmetric distributions of best gain- and cluster- threshold settings (*p*_gain_ < 0.05, *p*_clst_ < 10^−5^). Further significant improvements were possible by independently optimizing excitatory and inhibitory cluster thresholds (S4 Fig), but the gains were subtle and incurred substantially greater computation costs.

Despite the substantial improvements offered by gain thresholding and cluster-based analysis in our study, it is important to note these methods are not assumed to *eliminate* estimation noise in the STRF, only to *reduce* it relative to the raw STA. Further, our results do not suggest that these methods will necessarily outperform various alternative approaches developed for minimizing estimation noise in other preparations [32–34]. Considering the wide diversity and high dimensionality of stimulus and response spaces, best practices for receptive field estimation and noise correction are likely to vary across model organisms, sensory systems, and stations [75]. Indeed, as in previous reports, a minority of units in our study exhibited *decreased* prediction accuracy following each correction method, implying that no single method is likely to provide a universally ideal solution for a given neural population. Although a formal comparison between the current approaches and other previously published methods exceeded the scope of the present study, we explore below several apparent differences and common themes among these approaches below.

Alternatives to gain thresholding and cluster-based analysis typically incorporate knowledge about, and impose constraints upon, STRF structure. These include sparseness, smoothness, locality, and shape of the filters [32–34]. The current results are congruent with these approaches in demonstrating that the predictive quality of most receptive field estimates can be significantly improved by augmenting classic reverse correlation techniques with additional noise reduction steps. A second important parallel is that both gain thresholding and cluster-based methods are capable of improving prediction quality by only eliminating STRF pixels, thus resulting in relatively sparse STRFs similar to those produced by methods, such as automatic relevance determination, explicitly informed by this common property of STRFs [33–34]. A third parallel stems from the relative success of cluster-based correction approaches in our study, which suggest such methods are capable of improved balance between sensitivity and specificity over gain-thresholding techniques alone. Specifically, cluster analysis holds the advantage of potentially capturing low-gain (high-probability) pixels contiguous with high-gain (low-probability) pixels reflecting the true receptive field, while appropriately constraining false-positive pixels with similar low-gain values scattered throughout nonresponsive filter space. Consistent with this interpretation, mean prediction improvements were highest at the intersection of liberal *p*_gain_ and conservative *p*_clst_ settings (Fig 4A and 4B), and cross-validated threshold settings reiterated this trend (Fig 4C and 4D). Thus, similar to methods that explicitly bias receptive field locality such as automatic locality determination [33], cluster-based thresholds tend to enforce an element of spectrotemporal locality by implicitly favoring survival of individual pixels that contribute to the formation of local clusters.

From a general perspective, algorithms implemented in the current study for estimating best thresholds for individual units are conceptually similar to other methods in which various regularization parameters are specified through cross-validation. It is important to note, however, that such optimization algorithms are neither essential to gain-thresholding techniques nor cluster-based analysis. Indeed, fixed parameter approaches performed well in our study and may be preferable in some situations, e.g., where any potential decrease in accuracy is offset by a decrease in computation time afforded by obviation of the cross-validation step. In this regard, the current methods comprise a significant departure from those cited above, inasmuch as they require only noise distributions generated by null conditions, but are entirely agnostic as to the structure of the receptive fields themselves. A second practical difference is that the gain- and cluster-thresholding techniques are easily scalable to receptive fields of arbitrary resolution and dimensionality. The significance of this advantage can be appreciated when considering the alternative methods cited above require computing the stimulus covariance matrix defined by N × N stimulus dimensions (i.e., the number of elements in the STRF). Such requirements can quickly become computationally prohibitive for STRFs with large numbers of parameters, such as those evaluated in the current study, without additional discretization or dimensionality reduction steps. By avoiding these requirements, gain- and cluster-based correction methods make no compromises with respect to the dimensionality or resolution of the STRF estimates, and carry no risk of obscuring important fine spectral and temporal details. These advantages have likely contributed to the continued use of gain-thresholding techniques in auditory STRF experiments [e.g., 17,22,36–38], as well as the ubiquitous adoption of cluster-based analysis in the fMRI literature [50–54], which directly inspired the present analysis.

The outcomes of the present study have implications for understanding the relationship between prediction accuracy and the linearity of stimulus-response transforms. Highly predictable responses are generally assumed to imply linear transforms, whereas poorly predicted responses are thought to be dominated by nonlinear components [68–69]. As illustrated here, however, prediction correlations were heavily influenced by statistical correction choices, as well as the temporal resolution of the binned responses and predictions. Many additional methodological choices can influence prediction accuracy results, including the size (or fraction used) of the estimation and validation datasets [32,56,76], compensating for spike time jitter [77], smoothing response/prediction functions or receptive field kernels [16,72,76], excluding onset responses [72], and various technical details regarding stimulus representation approaches [78]. These factors raise important caveats for evaluating system response linearity [70], and especially for comparing results across studies obtained with different methodologies.

Accurate auditory STRF estimation comprises part of the more general problem of developing accurate and models of auditory cortical encoding [32]. Progress in these domains may be relevant to signal classification applications such as speech recognition algorithms [32], and are especially important for developing brain–computer interfaces and auditory cortical prostheses [79–80]. For these applications, MU signals are often particularly desirable since they do not require time- and computation-intensive spike sorting [81–82]. In this regard, our data suggest that correction procedures may be especially important, given that prediction improvements following STRF correction were more substantial for the MU data. STRFs obtained from local field potentials [19–20] and electrocorticography signals [21–23] are also directly relevant to such applications, and deserve future experimental attention with regard to statistical correction procedures.

In summary, STRF models have been widely adopted in sensory physiology as a means of representing stimulus features to which single neurons and cell populations are sensitive. However, it is important to bear in mind that STRF estimates will reflect accurate approximations of the true underlying receptive field only to the extent that they are obtained with proper methodological approaches. Among other factors, the legitimacy of STRF estimates critically depends on appropriate stimulus configurations and rigorous analytical methods [6]. Using predictive validity as a proxy for ‘ground truth’ regarding receptive field structure, the current and previous studies suggest that classic reverse correlation techniques can benefit considerably from evidence-based statistical correction procedures. In the present study, mean prediction accuracy improved by 82.3% in the MU data sample and by 40.3% in the SU data sample, reflecting an increase from capturing roughly one third to one half of the stimulus-driven variation in the PSTH. A considerable portion of the improvement observed in the present study was possible through simple gain-thresholding techniques common in the auditory STRF literature. However, we show that cluster-based analysis offers a conceptually straightforward extension of these techniques capable of yielding substantial additional predictive power without material changes in computational sophistication or assumptions about receptive field structure. Such changes in predictive validity imply structural differences among STRFs obtained with each correction approach, which were further confirmed by the finding that tBMF and sBMF parameter estimates changed by approximately 15%, on average, when non-conventional correction methods were employed. The changes and improvements obtained with these relatively simple correction methods are on par with those reported in previous studies, e.g., by improving estimation stimulus choices [23,66,83–84], stimulus representation methods [78], and encoding models [23,83,85], as well as changes incurred by contextual variables such as behavioral demands [86–87] and anesthetic state [20,88].

## Supporting information

S1 Dataset Experimental data. This dataset (.xlsx) contains the data points summarized in Figs 3–6 and 8 and S1–S4 Figs. Data for each figure are presented on separate sheets. Data are organized by subplot within sheets, and column headers are used to indicate data types and conditions.
(XLSX)

S1 Fig Excitatory and inhibitory STRF subfield ratios. **(A)** Most STRFs were dominated by excitatory responses, as indicated by the majority of the histogram mass falling above 1 (excitation = inhibition). The mean ratio between peak excitatory and inhibitory pixels was 1.83 for MU and 1.60 for SU data (indicated by markers). **(B)** Similarly, the mean ratio of summed excitatory and inhibitory cluster mass values (STRF gain threshold: *p*_gain_ < 0.05) was 1.21 for MU and 1.16 for SU data (indicated by markers).
(EPS)

S2 Fig Example data depicting results of independent excitatory and inhibitory gain- and cluster-mass thresholding procedures. Raw STAs were corrected with a continuum of excitatory and inhibitory gain and cluster-mass thresholds, expressed in terms of chance probability (*p*_gain{exc,inh}_, *p*_(gain,clst{exc,inh})_) determined by null STA values computed with randomly shifted spike times. The same five example units depicted in Fig 2 are used to illustrate the consequences of the gain thresholding (**A–E)** and cluster-based approaches (**F–J)**. For each subplot, corrected STAs are shown on the left, and plots of prediction accuracy at each tested threshold setting are shown on the right. Surface plots in (**F–J)** depicting prediction accuracy at each excitatory and inhibitory cluster threshold intersection are summarized for the gain threshold that yielded the best mean prediction accuracy (note: for plots in **(A–E)**, *p*_gain{exc,inh}_ = 10^0^ reflects the raw STA, and for plots in **(F–J)** *p*_clst{exc,inh}_ = 10^0^ corresponds to the STA corrected only with the gain threshold noted above the surface plots with no cluster thresholding). For many units, prediction accuracy depended more heavily on the excitatory gain threshold, reaching peak levels at narrow and broad ranges of excitatory and inhibitory thresholds, respectively **(A)**. In some cases, the reverse was true **(B–C)**. For other units, the consequences of excitatory and inhibitory gain correction on prediction accuracy were more symmetric **(D–E)**. The consequences of excitatory and inhibitory cluster thresholding were symmetric for most units **(F)**. Subtle asymmetries in the relative impact of each threshold were observed in some cases, favoring either a relatively narrow range of excitatory or inhibitory **(G–H)** cluster-mass thresholds. For other units, prediction increases were largely captured by basic gain thresholding, with negligible additional benefits observed for excitatory or inhibitory cluster-mass thresholding **(I–J)**. For a given gain threshold, changes in the STA were frequently not observed at each of the fine increments in cluster-mass threshold. E.g., the same clusters surviving a threshold of *p*_clst{exc}_ < 10^−7^ sometimes remained after thresholding at *p*_clst{exc}_ < 10^−9^, giving rise to coarse threshold-prediction functions characterized by local segments of identical prediction accuracy.
(EPS)

S3 Fig Summary of results from independent excitatory and inhibitory gain thresholding. **(A–B)** Mean prediction accuracy at each excitatory and inhibitory threshold intersection (*p*_gain{exc,inh}_) minus prediction accuracy obtained with raw STRFs (MU: *r* = 0.176, SU: *r* = 0.210). **(C–D)** Histograms of excitatory and inhibitory gain thresholds that yielded the highest prediction accuracy. For the MU sample, best *p*_gain{inh}_ thresholds were significantly more conservative than best *p*_gain{exc}_ thresholds, whereas a trend the same effect was insignificant in the SU sample (two-sample K-S tests). **(E–F)** Cumulative distribution functions of prediction accuracy obtained with raw STAs, STRFs corrected at the best *p*_gain_ settings (chosen for each unit by cross validation), STRFs corrected at a fixed setting (*p*_gain{exc,inh}_ < 0.01 applied uniformly across units), and STRFs corrected at the best *p*_gain{exc,inh}_ settings (chosen for each unit by cross validation). Inset bar plots represent mean +SEM prediction accuracy. Applying thresholds independently for excitatory and inhibitory time-frequency bins did not substantially change prediction accuracy. **(G)** Scatter plots of prediction accuracy for individual units at best *p*_gain{exc,inh}_ versus raw (left) and best *p*_gain_ (right). Red markers indicate the means. **(H)** Mean ± SEM prediction accuracy as a function of the temporal bin size for the PSTHs and predictions using the same correction approaches and color schemes as in **(E–F)**.
(EPS)

S4 Fig Summary of results from two-step (pixel-gain and cluster-mass) correction, with independent excitatory and inhibitory cluster mass thresholding. (**A–B)** Mean prediction accuracy at each excitatory and inhibitory cluster mass threshold intersection (*p*_(gain,clst{exc,inh})_), at the best *p*_gain_ for each unit, minus prediction accuracy obtained with raw STRFs (MU: *r* = 0.176, SU: *r* = 0.210). **C–D,** Histograms of excitatory and inhibitory cluster thresholds that yielded the highest prediction accuracy. No statistically reliable differences were observed between the best *p*_clst{exc}_ and best *p*_clst{inh}_ settings for either data type (two-sample K-S tests). **(E–F)** Cumulative distribution functions of prediction accuracy obtained with raw STAs, STRFs corrected at the best *p*_(gain,clst)_ settings (chosen for each unit by cross validation), STRFs corrected at a fixed setting (*p*_gain_ < 0.05, *p*_clst{exc}_ < 10^−5^, *p*_clst{inh}_ < 10^−5^ applied uniformly across units), and STRFs corrected at the best *p*_(gain,clst{exc,inh})_ settings (chosen for each unit by cross validation). Inset bar plots represent mean +SEM prediction accuracy. The best *p*_(gain,clst{exc,inh})_ settings yielded significant increases in prediction accuracy over the best *p*_(gain,clst)_ and fixed *p*_(gain,clst{exc,inh})_ methods, which were statistically equivalent to each other (Wilcoxon signed-rank tests). **(G)** Scatter plots of prediction accuracy for individual units at best *p*_(gain,clst{exc,inh})_ versus raw (left) and best *p*_(gain,clst)_ (right). Red markers indicate the means. **(H)** Mean ± SEM prediction accuracy as a function of the temporal bin size for the PSTHs and predictions using the same correction approaches and color schemes as in **(E–F)**.
(EPS)

## References

[1] Eggermont JJ, Johannesma PI, Aertsen AM. Reverse-correlation methods in auditory research. Quarterly Reviews of Biophysics. 1983; 16(03):341–414. 6366861 10.1017/s0033583500005126

[2] Kowalski N, Depireux DA, Shamma SA. Analysis of dynamic spectra in ferret primary auditory cortex. I. Characteristics of single-unit responses to moving ripple spectra. Journal of Neurophysiology. 1996; 76(5):3503–3523. 8930289 10.1152/jn.1996.76.5.3503

[3] Kowalski N, Depireux DA, Shamma SA. Analysis of dynamic spectra in ferret primary auditory cortex. II. Prediction of unit responses to arbitrary dynamic spectra. Journal of Neurophysiology. 1996; 76(5):3524–3534. 8930290 10.1152/jn.1996.76.5.3524

[4] Depireux DA, Simon JZ, Klein DJ, Shamma SA. Spectro-temporal response field characterization with dynamic ripples in ferret primary auditory cortex. Journal of Neurophysiology. 2001; 85(3):1220–1234. 11247991 10.1152/jn.2001.85.3.1220

[5] Atencio CA, Sharpee TO, Schreiner CE. Cooperative nonlinearities in auditory cortical neurons. Neuron. 2008; 58(6):956–966. doi: 10.1016/j.neuron.2008.04.026 18579084 10.1016/j.neuron.2008.04.026 PMC2535914

[6] Atencio CA, Schreiner CE. Stimulus choices for spike-triggered receptive field analysis In: Depireux DA, Elhilali M, editors. Handbook of modern techniques in auditory cortex. New York: Nova Biomedical; 2013 pp. 61–100.

[7] Wu MC, David SV, Gallant JL. Complete functional characterization of sensory neurons by system identification. Annual Reviews Neuroscience. 2006; 29:477–505. 10.1146/annurev.neuro.29.051605.113024 16776594

[8] Chichilnisky EJ. A simple white noise analysis of neuronal light responses. Network: Computation in Neural Systems. 2001; 12(2):199–213. 11405422

[9] Malone BJ, Kumar VR, Ringach DL. Dynamics of receptive field size in primary visual cortex. Journal of Neurophysiology. 2007; 97(1):407–414. doi: 10.1152/jn.00830.2006 17021020 10.1152/jn.00830.2006

[10] Ringach DL, Malone BJ. The operating point of the cortex: neurons as large deviation detectors. The Journal of Neuroscience. 2007; 27(29):7673–7683. doi: 10.1523/JNEUROSCI.1048-07.2007 17634362 10.1523/JNEUROSCI.1048-07.2007 PMC6672889

[11] Malone BJ, Ringach DL. Dynamics of tuning in the Fourier domain. Journal of Neurophysiology. 2008; 100(1):239–248. doi: 10.1152/jn.90273.2008 18480369 10.1152/jn.90273.2008 PMC2493484

[12] DiCarlo JJ, Johnson KO, Hsiao SS. Structure of receptive fields in area 3b of primary somatosensory cortex in the alert monkey. The Journal of Neuroscience. 1998; 18(7):2626–2645. 9502821 10.1523/JNEUROSCI.18-07-02626.1998 PMC6793113

[13] Sripati AP, Yoshioka T, Denchev P, Hsiao SS, Johnson KO. Spatiotemporal receptive fields of peripheral afferents and cortical area 3b and 1 neurons in the primate somatosensory system. The Journal of Neuroscience. 2006; 26(7):2101–2114. doi: 10.1523/JNEUROSCI.3720-05.2006 16481443 10.1523/JNEUROSCI.3720-05.2006 PMC1839048

[14] Machens CK, Wehr MS, Zador AM. Spectro-temporal receptive fields of subthreshold responses in auditory cortex In: Becker S, Thrun S, Obermayer K, editors. Advances in neural information processing systems. 15 ed. Cambridge: MIT; 2003 pp. 149–156.

[15] Machens CK, Wehr MS, Zador AM. Linearity of cortical receptive fields measured with natural sounds. The Journal of Neuroscience. 2004; 24(5):1089–1100. doi: 10.1523/JNEUROSCI.4445-03.2004 14762127 10.1523/JNEUROSCI.4445-03.2004 PMC6793584

[16] Kim G, Doupe A. Organized representation of spectrotemporal features in songbird auditory forebrain. The Journal of Neuroscience. 2011; 31(47):16977–16990. doi: 10.1523/JNEUROSCI.2003-11.2011 22114268 10.1523/JNEUROSCI.2003-11.2011 PMC3683074

[17] Rodríguez FA, Read HL, Escabí MA. Spectral and temporal modulation tradeoff in the inferior colliculus. Journal of Neurophysiology. 2010; 103(2):887–903. doi: 10.1152/jn.00813.2009 20018831 10.1152/jn.00813.2009 PMC2822687

[18] Valentine PA, Eggermont JJ. Stimulus dependence of spectro-temporal receptive fields in cat primary auditory cortex. Hearing Research. 2004; 196(1):119–133. 15464309 10.1016/j.heares.2004.05.011

[19] Eggermont JJ, Munguia R, Pienkowski M, Shaw G. Comparison of LFP-based and spike-based spectro-temporal receptive fields and cross-correlation in cat primary auditory cortex. PLoS ONE. 2011; 6(5):e20046 doi: 10.1371/journal.pone.0020046 21625385 10.1371/journal.pone.0020046 PMC3100317

[20] Montejo N, Noreña AJ. Dynamic representation of spectral edges in guinea pig primary auditory cortex. Journal of Neurophysiology. 2015; 113(7):2998–3012. doi: 10.1152/jn.00785.2014 25744885 10.1152/jn.00785.2014 PMC4416612

[21] Mesgarani N, Chang EF. Selective cortical representation of attended speaker in multi-talker speech perception. Nature. 2012; 485(7397):233–236. doi: 10.1038/nature11020 22522927 10.1038/nature11020 PMC3870007

[22] Escabí MA, Read HL, Viventi J, Kim DH, Higgins NC, Storage DA, et al A high-density, high-channel count, multiplexed μECoG array for auditory-cortex recordings. Journal of Neurophysiology. 2014; 112(6):1566–1583. doi: 10.1152/jn.00179.2013 24920021 10.1152/jn.00179.2013 PMC4137255

[23] Hullett PW, Hamilton LS, Mesgarani N, Schreiner CE, Chang EF. Human superior temporal gyrus organization of spectrotemporal modulation tuning derived from speech stimuli. The Journal of Neuroscience. 2016; 36(6):2014–2026. doi: 10.1523/JNEUROSCI.1779-15.2016 26865624 10.1523/JNEUROSCI.1779-15.2016 PMC4748082

[24] Schwartz O, Pillow JW, Rust NC, Simoncelli EP. Spike-triggered neural characterization. Journal of Vision. 2006; 6(4):484–507. doi: 10.1167/6.4.13 16889482 10.1167/6.4.13

[25] Sakai HM. White-noise analysis in neurophysiology. Physiological Reviews. 1992; 72(2):491–505. 1557430 10.1152/physrev.1992.72.2.491

[26] deCharms RC, Blake DT, Merzenich MM. Optimizing sound features for cortical neurons. Science. 1998; 280(5368):1439–1444. 9603734 10.1126/science.280.5368.1439

[27] Ringach D, Shapley R. Reverse correlation in neurophysiology. Cognitive Science. 2004; 28(2):147–166.

[28] Theunissen FE, Woolley S, Hsu A, Fremouw T. Methods for the analysis of auditory processing in the brain. Annals of the New York Academy of Sciences. 2004; 1016(1):187–207. 15313776 10.1196/annals.1298.020

[29] Theunissen FE, Sen K, Doupe AJ. Spectral-temporal receptive fields of nonlinear auditory neurons obtained using natural sounds. The Journal of Neuroscience. 2000; 20(6):2315–2331. 10704507 10.1523/JNEUROSCI.20-06-02315.2000 PMC6772498

[30] Sen K, Theunissen FE, Doupe AJ. Feature analysis of natural sounds in the songbird auditory forebrain. Journal of Neurophysiology. 2001; 86(3):1445–1458. 11535690 10.1152/jn.2001.86.3.1445

[31] Escabí MA, Schreiner CE. Nonlinear spectrotemporal sound analysis by neurons in the auditory midbrain. The Journal of Neuroscience. 2002; 22(10):4114–4131. 12019330 10.1523/JNEUROSCI.22-10-04114.2002 PMC6757655

[32] Thorson IL, Liénard J, David SV. The essential complexity of auditory receptive fields. PLoS Computational Biology 2015; 11(12):e1004628 doi: 10.1371/journal.pcbi.1004628 26683490 10.1371/journal.pcbi.1004628 PMC4684325

[33] Park M, Pillow JW. Receptive field inference with localized priors. PLoS Computational Biology. 2011; 7(10):e1002219 doi: 10.1371/journal.pcbi.1002219 22046110 10.1371/journal.pcbi.1002219 PMC3203052

[34] Sahani M, Linden JF. Evidence optimization techniques for estimating stimulus-response functions In: Becker S, Thrun S, Obermayer K, editors. Advances in neural information processing systems. 15 ed. Cambridge: MIT; 2003 pp. 317–324.

[35] Sharpee TO, Miller KD, Stryker MP. On the importance of static nonlinearity in estimating spatiotemporal neural filters with natural stimuli. Journal of Neurophysiology. 2008; 99(5):2496–2509. doi: 10.1152/jn.01397.2007 18353910 10.1152/jn.01397.2007 PMC2877595

[36] Atencio CA, Schreiner CE. Functional congruity in local auditory cortical microcircuits. Neuroscience. 2016; 316:402–419. doi: 10.1016/j.neuroscience.2015.12.057 26768399 10.1016/j.neuroscience.2015.12.057 PMC4728049

[37] Atencio CA, Shen V, Schreiner CE. Synchrony, connectivity, and functional similarity in auditory midbrain local circuits. Neuroscience. 2016; 335:30–53. doi: 10.1016/j.neuroscience.2016.08.024 27544405 10.1016/j.neuroscience.2016.08.024 PMC5031551

[38] Chen C, Rodriguez FC, Read HL, Escabí MA. Spectrotemporal sound preferences of neighboring inferior colliculus neurons: implications for local circuitry and processing. Frontiers in Neural Circuits. 2012; 6:62 doi: 10.3389/fncir.2012.00062 23060750 10.3389/fncir.2012.00062 PMC3461703

[39] Escabí MA, Miller LM, Read HL, Schreiner CE. Naturalistic auditory contrast improves spectrotemporal coding in the cat inferior colliculus. The Journal of Neuroscience. 2003; 23(37):11489–11504. 14684853 10.1523/JNEUROSCI.23-37-11489.2003 PMC6740949

[40] Andoni S, Li N, Pollak GD. Spectrotemporal receptive fields in the inferior colliculus revealing selectivity for spectral motion in conspecific vocalizations. The Journal of Neuroscience. 2007; 27(18):4882–4893. doi: 10.1523/JNEUROSCI.4342-06.2007 17475796 10.1523/JNEUROSCI.4342-06.2007 PMC6672083

[41] Bartlett EL, Sadagopan S, Wang X. Fine frequency tuning in monkey auditory cortex and thalamus. Journal of Neurophysiology. 2011; 106(2):849–859. doi: 10.1152/jn.00559.2010 21613589 10.1152/jn.00559.2010 PMC3154823

[42] Bitterman Y, Mukamel R, Malach R, Fried I, Nelken I. Ultra-fine frequency tuning revealed in single neurons of human auditory cortex. Nature. 2008; 451(7175):197–201. doi: 10.1038/nature06476 18185589 10.1038/nature06476 PMC2676858

[43] Joris PX, Bergevin C, Kalluri R, Mc Laughlin M, Michelet P, van der Heijden, et al Frequency selectivity in Old-World monkeys corroborates sharp cochlear tuning in humans. Proceedings of the National Academy of Sciences USA. 2011; 108(42):17516–17520. 10.1073/pnas.1105867108 PMC3198376 21987783

[44] Garcia-Lazaro JA, Belliveau LA, Lesica NA. Independent population coding of speech with sub-millisecond precision. The Journal of Neuroscience. 2013; 33(49):19362–19372. doi: 10.1523/JNEUROSCI.3711-13.2013 24305831 10.1523/JNEUROSCI.3711-13.2013 PMC3850048

[45] Kayser C, Logothetis NK, Panzeri S. Millisecond encoding precision of auditory cortex neurons. Proceedings of the National Academy of Sciences USA. 2010; 107(39):16976–16981. 10.1073/pnas.1012656107 PMC2947890 20837521

[46] Malone BJ, Scott BH, Semple MN. Encoding frequency contrast in primate auditory cortex. Journal of Neurophysiology. 2014; 111(11):2244–2263. 24598525 10.1152/jn.00878.2013 PMC4097870

[47] Malone BJ, Scott BH, Semple MN. Temporal codes for amplitude contrast in auditory cortex. The Journal of Neuroscience. 2010; 30(2):767–784. doi: 10.1523/JNEUROSCI.4170-09.2010 20071542 10.1523/JNEUROSCI.4170-09.2010 PMC3551278

[48] Yang Y, DeWeese MR, Otazu GH, Zador AM. Millisecond-scale differences in neural activity in auditory cortex can drive decisions. Nature Neuroscience. 2008; 11(11):1262–1263. doi: 10.1038/nn.2211 18849984 10.1038/nn.2211 PMC4062077

[49] Atencio CA, Schreiner CE. Spectrotemporal processing in spectral tuning modules of cat primary auditory cortex. PLoS ONE. 2012; 7(2):e31537 doi: 10.1371/journal.pone.0031537 22384036 10.1371/journal.pone.0031537 PMC3288040

[50] Heller R, Stanley D, Yekutieli D, Rubin N, Benjamini Y. Cluster-based analysis of FMRI data. Neuroimage. 2006; 33(2):599–608. doi: 10.1016/j.neuroimage.2006.04.233 16952467 10.1016/j.neuroimage.2006.04.233

[51] Bennett CM, Wolford GL, Miller MB. The principled control of false positives in neuroimaging. Social Cognitive and Affective Neuroscience. 2009; 4(4):417–422. doi: 10.1093/scan/nsp053 20042432 10.1093/scan/nsp053 PMC2799957

[52] Woo CW, Krishnan A, Wager TD. Cluster-extent based thresholding in fMRI analyses: pitfalls and recommendations. Neuroimage. 2014; 91:412–419. doi: 10.1016/j.neuroimage.2013.12.058 24412399 10.1016/j.neuroimage.2013.12.058 PMC4214144

[53] Auer T, Schweizer R, Frahm J. An iterative two-threshold analysis for single-subject functional MRI of the human brain. European Radiology. 2011; 21(11):2369–2387. doi: 10.1007/s00330-011-2184-5 21710268 10.1007/s00330-011-2184-5

[54] Ing A, Schwarzbauer C. Cluster size statistic and cluster mass statistic: two novel methods for identifying changes in functional connectivity between groups or conditions. PLoS ONE. 2014; 9(6):e98697 doi: 10.1371/journal.pone.0098697 24906136 10.1371/journal.pone.0098697 PMC4048154

[55] Natan RG, Carruthers IM, Mwilambwe-Tshilobo L, Geffen MN. Gain control in the auditory cortex evoked by changing temporal correlation of sounds. Cerebral Cortex. 2016; 27(3):2385–2402. 10.1093/cercor/bhw083 PMC6059244 27095823

[56] Honey C, Schnupp J. Neural resolution of formant frequencies in the primary auditory cortex of rats. PLoS ONE. 2015; 10(8):e0134078 doi: 10.1371/journal.pone.0134078 26252382 10.1371/journal.pone.0134078 PMC4529216

[57] David SV, Gallant JL. Predicting neuronal responses during natural vision. Network: Computation in Neural Systems. 2005; 16(2–3):239–260. 10.1080/09548980500464030 16411498

[58] Malone BJ, Beitel RE, Vollmer M, Heiser MA, Schreiner CE. Spectral context affects temporal processing in awake auditory cortex. The Journal of Neuroscience. 2013; 33(22):9431–9450. doi: 10.1523/JNEUROSCI.3073-12.2013 23719811 10.1523/JNEUROSCI.3073-12.2013 PMC3829847

[59] Malone BJ, Beitel RE, Vollmer M, Heiser MA, Schreiner CE. Modulation-frequency-specific adaptation in awake auditory cortex. The Journal of Neuroscience. 2015; 35(15):5904–5916. doi: 10.1523/JNEUROSCI.4833-14.2015 25878263 10.1523/JNEUROSCI.4833-14.2015 PMC4397592

[60] Cheung SW, Bedenbaugh PH, Nagarajan SS, Schreiner CE. Functional organization of squirrel monkey primary auditory cortex: responses to pure tones. Journal of Neurophysiology. 2001; 85(4):1732–1749. 11287495 10.1152/jn.2001.85.4.1732

[61] Godey B, Atencio CA, Bonham BH, Schreiner CE, Cheung SW. Functional organization of squirrel monkey primary auditory cortex: responses to frequency-modulation sweeps. Journal of Neurophysiology. 2005; 94(2):1299–1311. doi: 10.1152/jn.00950.2004 16061492 10.1152/jn.00950.2004

[62] Atencio CA, Blake DT, Strata F, Cheung SW, Merzenich MM, Schreiner CE. Frequency-modulation encoding in the primary auditory cortex of the awake owl monkey. Journal of Neurophysiology. 2007; 98(4):2182–2195. doi: 10.1152/jn.00394.2007 17699695 10.1152/jn.00394.2007

[63] Bialek W, van Steveninck RR. Features and dimensions: Motion estimation in fly vision. arXiv. 2005; preprint q-bio/0505003.

[64] Atencio CA, Schreiner CE. Columnar connectivity and laminar processing in cat primary auditory cortex. PLoS ONE. 2010; 5(3):e9521 doi: 10.1371/journal.pone.0009521 20209092 10.1371/journal.pone.0009521 PMC2831079

[65] Malone BJ, Scott BH, Semple MN. Dynamic amplitude coding in the auditory cortex of awake rhesus macaques. Journal of Neurophysiology. 2007; 98(3):1451–1474. doi: 10.1152/jn.01203.2006 17615123 10.1152/jn.01203.2006

[66] Atencio CA, Schreiner CE. Laminar diversity of dynamic sound processing in cat primary auditory cortex. Journal of Neurophysiology. 2010; 103(1):192–205. doi: 10.1152/jn.00624.2009 19864440 10.1152/jn.00624.2009 PMC2807218

[67] David SV, Mesgarani N, Fritz JB, Shamma SA. Rapid synaptic depression explains nonlinear modulation of spectro-temporal tuning in primary auditory cortex by natural stimuli. The Journal of Neuroscience. 2009; 29(11):3374–3386. doi: 10.1523/JNEUROSCI.5249-08.2009 19295144 10.1523/JNEUROSCI.5249-08.2009 PMC2774136

[68] Massoudi R, Van Wanrooij MM, Versnel H, Van Opstal AJ. Spectrotemporal response properties of core auditory cortex neurons in awake monkey. PLoS ONE. 2015; 10(2):e0116118 doi: 10.1371/journal.pone.0116118 25680187 10.1371/journal.pone.0116118 PMC4332665

[69] Versnel H, Zwiers MP, van Opstal AJ. Spectrotemporal response properties of inferior colliculus neurons in alert monkey. The Journal of Neuroscience. 2009; 29(31):9725–9739. doi: 10.1523/JNEUROSCI.5459-08.2009 19657026 10.1523/JNEUROSCI.5459-08.2009 PMC6666592

[70] Atencio CA, Sharpee TO, Schreiner CE. Receptive field dimensionality increases from the auditory midbrain to cortex. Journal of Neurophysiology. 2012; 107(10):2594–2603. doi: 10.1152/jn.01025.2011 22323634 10.1152/jn.01025.2011 PMC3362274

[71] Sahani M, Linden JF. How linear are auditory cortical responses? In: Becker S, Thrun S, Obermayer K, editors. Advances in neural information processing systems. 15 ed. Cambridge: MIT; 2003 pp. 125–132.

[72] Hsu A, Borst A, Theunissen FE. Quantifying variability in neural responses and its application for the validation of model predictions. Network: Computation in Neural Systems. 2004; 15(2):91–109. 15214701

[73] Vintch B, Movshon JA, Simoncelli EP. A convolutional subunit model for neuronal responses in macaque V1. The Journal of Neuroscience. 2015; 35(44):14829–14841. doi: 10.1523/JNEUROSCI.2815-13.2015 26538653 10.1523/JNEUROSCI.2815-13.2015 PMC4635132

[74] Sherrington CS. The integrative action of the nervous system New York: Scribner; 1906.

[75] Pillow JW, Simoncelli EP. Dimensionality reduction in neural models: an information-theoretic generalization of spike-triggered average and covariance analysis. Journal of Vision. 2006; 6(4): 414–428. doi: 10.1167/6.4.9 16889478 10.1167/6.4.9

[76] Nagel KI, Doupe AJ. Temporal processing and adaptation in the songbird auditory forebrain. Neuron. 2006; 51(6):845–859. doi: 10.1016/j.neuron.2006.08.030 16982428 10.1016/j.neuron.2006.08.030

[77] Gollisch T. Estimating receptive fields in the presence of spike-time jitter. Network: Computation in Neural Systems. 2006; 17(2):103–129. 10.1080/09548980600569670 16818393

[78] Gill P, Zhang J, Woolley SM, Fremouw T, Theunissen FE. Sound representation methods for spectro-temporal receptive field estimation. Journal of Computational Neuroscience. 2006; 21(1):5–20. doi: 10.1007/s10827-006-7059-4 16633939 10.1007/s10827-006-7059-4

[79] Mesgarani N, David SV, Fritz JB, Shamma SA. Influence of context and behavior on stimulus reconstruction from neural activity in primary auditory cortex. Journal of Neurophysiology. 2009; 102(6):3329–3339. doi: 10.1152/jn.91128.2008 19759321 10.1152/jn.91128.2008 PMC2804432

[80] Pasley BN, David SV, Mesgarani N, Flinker A, Shamma SA, et al Reconstructing speech from human auditory cortex. PLoS Biology. 2012; 10(1):e1001251 doi: 10.1371/journal.pbio.1001251 22303281 10.1371/journal.pbio.1001251 PMC3269422

[81] Todorova S, Sadtler P, Batista A, Chase S, Ventura V. To sort or not to sort: the impact of spike-sorting on neural decoding performance. Journal of Neural Engineering. 2014; 11(5):056005 doi: 10.1088/1741-2560/11/5/056005 25082508 10.1088/1741-2560/11/5/056005 PMC4454741

[82] Oby ER, Perel S, Sadtler PT, Ruff DA, Mischel JL, et al Extracellular voltage threshold settings can be tuned for optimal encoding of movement and stimulus parameters. Journal of Neural Engineering. 2016; 13(3):036009 doi: 10.1088/1741-2560/13/3/036009 27097901 10.1088/1741-2560/13/3/036009 PMC5931220

[83] Calabrese A, Schumacher JW, Schneider DM, Paninski L, Woolley SM. A generalized linear model for estimating spectrotemporal receptive fields from responses to natural sounds. PLoS ONE. 2011; 6(1):e16104 doi: 10.1371/journal.pone.0016104 21264310 10.1371/journal.pone.0016104 PMC3019175

[84] Schneider DM, Woolley SM. Extra-classical tuning predicts stimulus-dependent receptive fields in auditory neurons. The Journal of Neuroscience. 2011; 31(33):11867–11878. doi: 10.1523/JNEUROSCI.5790-10.2011 21849547 10.1523/JNEUROSCI.5790-10.2011 PMC3164972

[85] David SV, Shamma SA. Integration over multiple timescales in primary auditory cortex. The Journal of Neuroscience. 2013; 33(49):19154–19166. doi: 10.1523/JNEUROSCI.2270-13.2013 24305812 10.1523/JNEUROSCI.2270-13.2013 PMC3850039

[86] Fritz J, Shamma S, Elhilali M, Klein D. Rapid task-related plasticity of spectrotemporal receptive fields in primary auditory cortex. Nature Neuroscience. 2003; 6(11):1216–1223. doi: 10.1038/nn1141 14583754 10.1038/nn1141

[87] Fritz JB, Elhilali M, Shamma SA. Differential dynamic plasticity of A1 receptive fields during multiple spectral tasks. The Journal of Neuroscience. 2005; 25(33):7623–7635. doi: 10.1523/JNEUROSCI.1318-05.2005 16107649 10.1523/JNEUROSCI.1318-05.2005 PMC6725393

[88] Schumacher JW, Schneider DM, Woolley SM. Anesthetic state modulates excitability but not spectral tuning or neural discrimination in single auditory midbrain neurons. Journal of Neurophysiology. 2011; 106(2):500–514. doi: 10.1152/jn.01072.2010 21543752 10.1152/jn.01072.2010 PMC3154814

